# Preparation and Evaluation of PVDF-HFP-Based Gel Electrolyte for Ge-Sensitized Thermal Cell

**DOI:** 10.3390/polym16121732

**Published:** 2024-06-18

**Authors:** Yadong Chai, Sachiko Matsushita

**Affiliations:** 1 Department of Materials Science and Engineering, Tokyo Institute of Technology, 4259 Nagatsuta-cho, Midori-ku, Yokohama 226-8501, Kanagawa, Japan; chai.y.ac@m.titech.ac.jp; 2 elleThermo, Ltd., 3-3-6 Shibaura, Minato-ku, Tokyo 108-0023, Japan

**Keywords:** gel polymer electrolyte, thermal energy conversion, thermoelectric material, semiconductor, redox reaction, renewable energy

## Abstract

The semiconductor-sensitized thermal cell (STC) is a new thermoelectric conversion technology. The development of nonliquid electrolytes is the top priority for the practical application of the STC. In this study, a novel gel polymer electrolyte (PH-based GPE) composed of poly(vinylidenefluoride-*co*-hexafluoropropylene) (PH), 1-Methyl-2-pyrrolidone (NMP), and Cu ions was synthesized and applied to the STC system. The PH-based GPE synthesized at 45 °C showed higher open-circuit voltage (−0.3 V), short-circuit current density (59 μA cm^−2^) and diffusion coefficient (7.82 × 10^−12^ m^2^ s^−1^), indicating that a well-balanced structure among the NMP molecules was formed to generate a high-efficiency conduction path of the Cu ions. Moreover, the ion diffusion lengths decreased with decreasing content rates of NMP for the PH-based GPEs, indicating that the NMP plays an important role in the diffusion of Cu ions. Furthermore, the activation energy was calculated to be 107 kJ mol^−1^, and that was smaller compared to 150 kJ mol^−1^ for the poly(ethylene glycol)-based liquid electrolyte. These results play an important reference role in the development of electrolytes for STC systems. At the same time, they also provide a new avenue and reference indicator for the synthesis of high-performance and safe GPEs.

## 1. Introduction

The consumption of fossil energy such as oil and coal is continuing to increase with global population growth and economic growth. At the same time, climate change caused by greenhouse gas emissions would cause an unpredictable impact on the living environment of animals and plants. Thus, clean energy such as solar, wind, and geothermal power is expected to be used to reduce the burden on the environment. Compared with the unstable power generation of solar energy and wind power, thermal energy such as geothermal energy is a clean and stable energy supply method. In order to effectively utilize thermal energy, conversion technology of thermal energy is indispensable. The typical conversion technologies of thermal energy are geothermal power generation by turning the turbine [1] and thermoelectric energy conversion using the Seebeck effect [2,3]. The geothermal power generation would not emit greenhouse gases and can produce stable power generation. However, the development area competes with protected areas such as national parks and the hot spring industry, and the huge cost and risk involved in construction is a challenge. On the other hand, thermoelectric energy conversion using the Seebeck effect utilizes the number of excited carriers in a material due to temperature difference. By creating a temperature difference between the two ends of a certain material, carriers can flow from the high-temperature part to the low-temperature part and generate electric power. However, it is necessary to incorporate a low-temperature section to generate electricity, which makes the structure of the device complex and prevents its practical applications. Therefore, an effective and practical thermal energy conversion technology is needed.

The semiconductor-sensitized thermal cell (STC) has been developed to establish a new thermoelectric conversion technology in our group [4,5,6,7,8,9]. In detail, when thermal energy is applied to the semiconductor, thermally excited charges (electrons and holes) are generated in the semiconductor, and then they reduce and oxidize electrolyte ions. Subsequently, thermal electricity generation was achieved through diffusion and self-electron exchange of electrolyte ions. According to our previous reports [7,8,9], electricity is generated at 80 °C in the STC system using an *n*-Si/Ge substrate as the working electrode, a fluoride-doped tin oxide (FTO) substrate as the counter electrode, and Cu^2+^ and Cu^+^ electrolyte ions dissolved in poly(ethylene glycol) (PEG600) as the liquid electrolyte. This electricity generation is terminated when the diffusion and supply of electrolyte ions cannot keep up with the charge transfer at the electrode/electrolyte interface. Moreover, it has been found that the cell can be discharged again by leaving the discharged cell in an open-circuit state because ions can be rediffused in the electrolyte during the open-circuit process. However, the shape change of the PEG600 is easy to generate because it is a liquid polymer with fluidity. Thus, the contact area between the PEG600-based liquid electrolyte and the electrode is easy to change, resulting in unstable generation. The freedom of design is also limited. In addition, the liquid polymer electrolyte has the risk of leakage during long-term charge/discharge cycles in the STC system. Therefore, it is necessary to develop a more sustainable and safer electrolyte for STC system.

Compared with the liquid polymer electrolytes, solid polymer electrolytes are generally more stable against external factors [10], which have been studied as promising alternatives to liquid polymer electrolytes [11,12,13]. Solid polymer electrolytes offer several advantages, including nonflammable properties, no risk of internal short circuits, and no leakage issues. However, solid polymer electrolytes also have some disadvantages such as low ionic conductivity (10^−8^ S cm^−1^) and weak electrolyte–electrode interface [14,15,16]. In recent years, various strategies and efforts have been undertaken to improve the electrochemical performance of electrolytes. In particular, gel polymer electrolytes (GPEs) have received considerable attention because they combine the diffusion properties of liquids and the physical stability of solids [17,18,19]. Moreover, GPEs are able to overcome the disadvantages of liquid and solid polymer electrolytes, showing higher electrochemical stability and more stable interface with the electrodes [20,21]. The polymers used as matrixes for GPEs include Poly(acrylonitrile) (PAN) [22], poly(methyl methacrylate) (PMMA) [23], poly(ethylene oxide) (PEO) [24], poly(vinylidenedifluoride) (PVDF) [25], and poly(vinylidenefluoride-*co*-hexafluoropropylene) (PVDF-HFP) [26]. Among them, PVDF-HFP is a potential choice for GPE matrixes due to their high dielectric constants, prominent mechanical strength, electrochemical stability, and the presence of strong electron-withdrawing C–F bonds. PVDF-HFP consists of crystalline phases and amorphous phases, and its crystallinity decreases with increasing content of amorphous HFP. Thus, compared with highly crystalline PVDF, PVDF-HFP has better properties, such as easier plasticizing, higher solubility in organic solvents, and better electrochemical stability due to the combination of HFP [27,28]. For example, there was a report that a GPE composed of PVDF-HFP as matrix and N-methyl-2-pyrrolidone (NMP) as organic solvent was prepared through the solution-casting method [29]. It demonstrates an excellent interfacial stability with the electrode and a high ionic conductivity (7.24 × 10^−4^ S cm^−1^). Thus, it is worth researching the electrochemical performance of the GPE applied to the STC system.

In this study, a novel gel polymer electrolyte composed of PVDF-HFP (PH), NMP (PH-based GPE), and Cu ions was developed. It is the first report using PH-based GPE in the STC system. In order to determine the optimal gelation temperature condition, the chemical properties and electrochemical performance of the PH-based GPE synthesized under different temperatures were evaluated. We are devoted to finding the suitable synthesis conditions of the PH-based GPE with high performance for the STC system.

## 2. Materials and Methods

### 2.1. Chemicals

The *n*-Si/Ge substrate (Ge layer, 2 μm) was manufactured by Tohnic Corp., Chigasaki, Japan. A Cr layer (20 nm) was deposited on the *n*-Si wafer (0.02 Ω cm, 15 × 25 × 0.5 mm) to improve the adhesion of the *n*-Si/Ge interface. The resistivities of *n*-Si and Ge sides were 3.18 and 3.27 Ω/sq, which were measured by a four-probe resistivity meter (Loresta GP MCP-T600, Mitsubishi Chemical Corp., Tokyo, Japan) at room temperature. The fluorine-doped tin oxide (FTO, sheet resistivity: 10 Ω, 25 × 25 × 1.1 mm) substrate was manufactured by Peccell Technologies, Inc. Yokohama, Japan. Ethanol, hydrofluoric acid (HF), copper(I) chloride (CuCl), and copper(II) chloride (CuCl_2_) were purchased from FUJIFILM Wako Pure Chemical Corp., Osaka, Japan. 1-Methyl-2-pyrrolidone (NMP) was purchased from Tokyo Chemical Industry Co., Ltd., Tokyo, Japan. Poly(vinylidene fluoride-*co*-hexafluoropropylene) (PH) was purchased from Sigma-Aldrich Co. LLC, St. Louis, MO, USA. Electric insulation double-sided tape (thickness: 85 µm) was purchased from Chukoh Chemical Industries, Ltd., Tokyo, Japan. Sealing material was purchased from Momentive Performance Materials Worldwide LLC, New York, NY, USA.

### 2.2. Electrodes

The *n*-Si/Ge substrate as working electrode was ultrasonically washed in ethanol for 5 min and then immersed with 5% HF for 5 min to remove the oxide layer on the Ge surface, and washed with ultrapure water and promptly introduced into the purge-type glove box with a gas recycle purification system (Miwa Manufacturing Co., Ltd., Osaka, Japan). The glove box was hermetically filled with Ar gas (the oxygen concentration was 0.2~0.5 ppm). On the other hand, the FTO substrate as counter electrode was also ultrasonically washed in ethanol for 5 min and then introduced into the glove box. FTO is known for its excellent cost performance because it is cheaper than indium tin oxide. Moreover, it has chemically stable, high-temperature resistance and optical transparency. Thus, FTO transparent electrodes are used as counter electrodes to conveniently observe the state of the electrolyte in the STC system.

### 2.3. Electrolyte

The electrolyte solution was prepared in the glove box by mixing NMP (4.0 g) and PH (1.0 g), which was stirred at 50 °C for 6 h. Subsequently, CuCl (0.16 g) and CuCl_2_ (0.21 g) were added and stirred at 50 °C for 12 h to obtain the electrolyte solution (PH-NMP-Cu). The Cu ion concentration was determined by referring to the report about electrolytes of lithium metal batteries [29]. As for reference samples, a reference solution (NMP-Cu) was prepared by mixing NMP, CuCl and CuCl_2_, and another reference solution (PH-NMP) was prepared by mixing PH and NMP under the same conditions as above.

### 2.4. Cell Assembly

**Figure 1:**
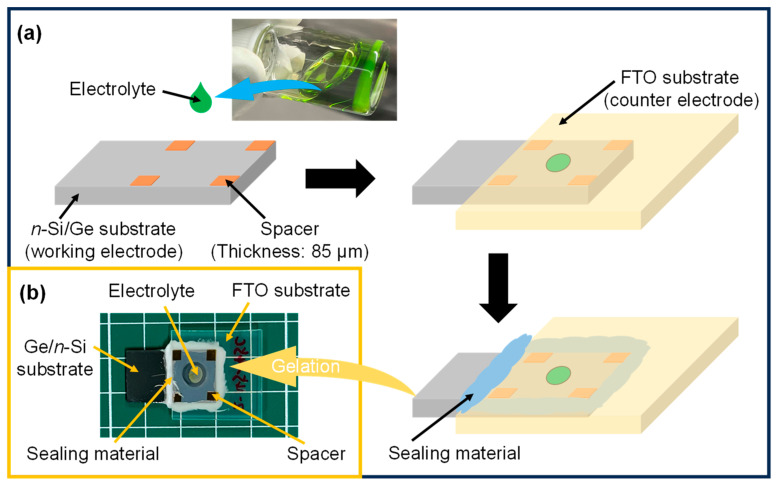
(**a**) Illustration of the cell assembly procedure and (**b**) digital camera image of the assembled cell.

The cell assembly procedure is shown in Figure 1, which was also conducted in the glove box. In detail, in order to control the distance between the FTO and *n*-Si/Ge electrodes, the electric insulation double-sided tape was pasted on the edges of the FTO substrate as a spacer. After that, 3.0 μL of the electrolyte solution (PH-NMP-Cu) was dropped onto the center of the FTO substrate, and then the *n*-Si/Ge substrate was placed to cover the electrolyte solution where the electrolyte was sandwiched between the FTO and *n*-Si/Ge electrodes. Subsequently, the gap between the electrodes was sealed by the sealing material to obtain the assembled cell. Finally, the cells were dried in a constant-temperature drying oven at different temperatures (30, 35, 40, 45, 50, 55, and 60 °C) for 18 h, respectively, to control the gelation temperatures of the electrolytes. As for the cells sealed by the sealing material and dried at different temperatures for 18 h, colors and morphologies of the electrolytes were confirmed to be unchanged, even leaving them in the air more than one month.

### 2.5. Characterization

Infrared spectra were measured by a Fourier transform infrared (FT-IR) spectroscopic method with attenuated total reflectance (FT/IR-4X, JASCO Corp., Tokyo, Japan). All the spectra were measured in the range of 2000~400 cm^−1^ with an accumulation time of 256 and a spectral resolution of 2 cm^−1^. The integrated area of the C=O absorption band due to NMP between 1740 and 1540 cm^−1^ and that of the C-CF_2_ absorption band due to PVDF-HFP between 900 and 860 cm^−1^ were calculated and named *A*_1_ and *A*_2_, respectively, to calculate the band ratio of *A*_1_ to *A*_2_ (*A*_1_/*A*_2_). Here, the integrated area was obtained by fitting a Gaussian function using the attached software (Spectrum Manager Ver 2.5, JASCO Corp., Tokyo, Japan). The content rates of NMP (wt%) in the electrolytes gelled at different temperatures were calculated through Equation (1). Here, the content rate of NMP in the PH-NMP-Cu solution was calculated to be 74.5 wt% (Experimental Procedure S1) and *T* represents the gelation temperature.

(1) $$\mathrm{Content}\;\mathrm{rate}\;\mathrm{of}\;\mathrm{NMP}=\frac{74.5\times {\left(A_{1}∕A_{2}\right)}_{T}}{{\left(A_{1}∕A_{2}\right)}_{\mathrm{PH}-\mathrm{NMP}-\mathrm{Cu}}}$$

Microphotographs of the electrolytes were taken with a reflected light microscope (BX51TRF, OLYMPUS Corp., Tokyo, Japan). All of the following electrochemical measurements were performed using a VSP-300 instrument (Bio-Logic, Seyssinet-Pariset, France). The cells were connected to the VSP-300 and set in ovens where the measuring temperatures were the same as their gelation temperatures (30, 35, 40, 45, 50, 55, and 60 °C). The cyclic voltammetry (CV) curves were measured at the scan rate of 10 mV/s. Moreover, in order to perform the long-term measurements, chronopotentiometry (CP) was also performed at a constant current of 200 nA. Temperature dependence of short-circuit current density (Arrhenius plot) was measured to calculate activation energy through Equation (2) [30]. Here, *I*_sc_ (A cm^−2^) is the short circuit current density which was calculated by dividing the short-circuit current through the contact area between the gelled electrolyte and the electrode; the area was measured by a program named ImageJ where the part lacking integrity was not included in the calculation; *E_a_* (kJ mol^−1^) is the activation energy; *R* (J K^−1^ mol^−1^) is the gas constant; *T* (K) is the temperature; and *K* is the constant independent of temperature (frequency factor).

(2) $$\ln I_{\mathrm{sc}}=-\frac{E_{a}}{R\;}\times \frac{1000}{T}+\ln K$$

The electrolytes were measured with a UV–Vis spectrophotometer (V-770, JASCO Corp.). The absorbance in the wavelength range between 600 and 1400 nm was measured under the bandwidth of 2 nm and scanning speed of 400 nm min^−1^.

The CV curves with different scan rates changing from 10 to 90 mV s^−1^ were measured to calculate the diffusion coefficients through Equation (3) [31,32,33]. The symmetric cells were used for measurement, in which the electrolyte was sandwiched between the FTO and FTO electrodes. This equation is derived from the similar adsorption process theory at the electrode/electrolyte interface, in which the charge transfer at the interface is fast enough compared with the ion diffusion. Here, *I_p_* (A) is the peak current, *n* is the number of reaction electrons, *A* (cm^2^) is the contact area between electrolyte and electrode, *D* (cm^2^ s^−1^) is the diffusion coefficient which is average value of diffusion coefficient of oxidant and reductant, *c* (mol cm^−3^) is ion concentration, and *v* (V s^−1^) is scan rate.

(3) $$I_{p}=(2.69\times 10^{5}){\;n}^{3/2}A\;D^{1/2}\;c\;v^{1/2}$$

Potentiostat electrochemical impedance spectroscopy (PEIS) was also performed. The symmetric cells were used for PEIS measurement, in which the electrolyte was sandwiched between the FTO and FTO electrodes. In PEIS, the amplitude of the alternative current was set to 10 mV. The frequency range was from 7 MHz to 50 mHz. ZView software (Ver 3.5i, Scribner Associates Inc. Southern Pines, U.S.) was employed for the fitting to calculate the electrolyte resistance (*R_e_* (Ω)) and Warburg element representative of ion diffusion (*W* (s)). Moreover, the ion conductivities (*σ* (cm^−1^ Ω^−1^)) and the ion diffusion lengths (*l* (cm)) were calculated through Equations (4) [34] and (5) [35], respectively. Here, *d* (cm) is the distance between the electrodes, which is 85 × 10^−4^ cm.

(4) $$\sigma =d/(A\;R_{e})$$

(5) $$l={\left(D\;W\right)}^{1/2}$$

## 3. Results

### 3.1. Chemical Bonding States and Morphologies of GPEs

**Figure 2:**
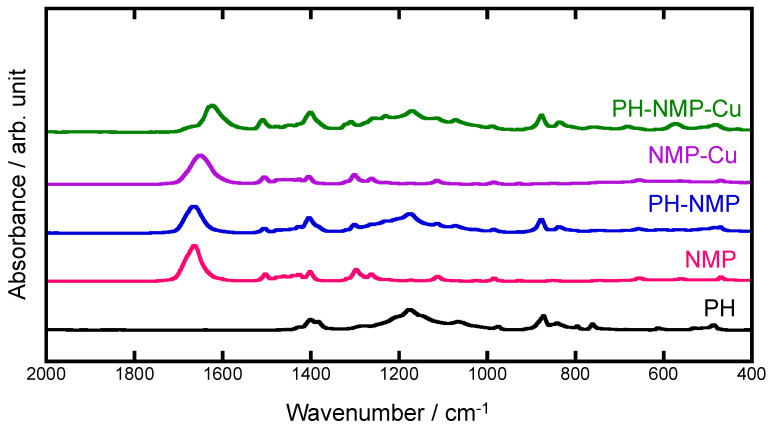
FT-IR spectra of (PVDF-HFP) PH, NMP, PH-NMP, NMP-Cu, and PH-NMP-Cu.

**Chemical bonding states of electrolyte and reference solutions.** FT-IR spectra of PH-NMP-Cu, NMP-Cu, PH-NMP, pure NMP, and PH are shown in Figure 2. The characteristic absorption bands due to NMP and PH were observed for PH-NMP-Cu electrolyte solution [36,37,38,39]. As for pure NMP, the C=O stretching vibration mode appeared at 1664 cm^−1^ [36]. It is worth noting that the C=O stretching vibrations of PH-NMP-Cu and NMP-Cu solutions appeared at 1623 and 1651 cm^−1^, which were shifted to the lower wavenumber side compared with that of the NMP solution. It indicated that there are chemical interactions between Cu ions and electronegative C=O of NMP [29,36], in which the bonding electrons existing in the C=O were partially transferred to the Cu resulting in reduction in the bond energy and shift of the stretching vibration region for C=O. The adsorption peak due to the C=O stretching vibrations of PH-NMP-Cu shifted to lower wavenumber side than that of NMP-Cu, suggesting that the proportion of free NMP was lower and the proportion of NMP combined with Cu was higher for the PH-NMP-Cu.

**Figure 3:**
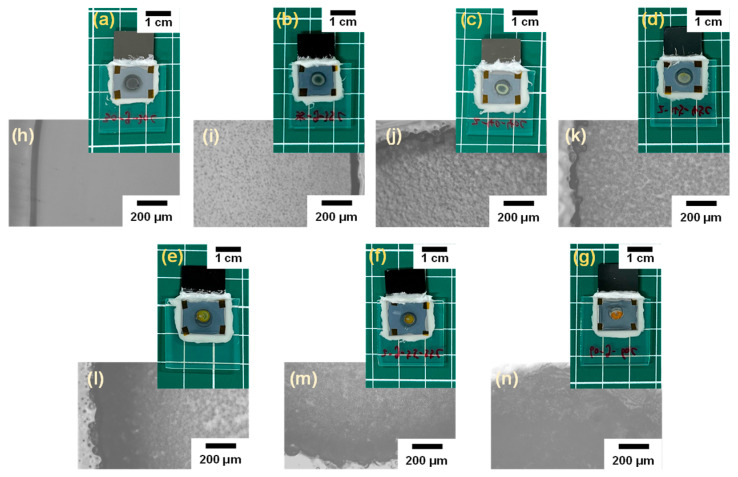
(**a**–**g**) Digital camera images of the fabricated cells and (**h**–**n**) microphotographs of the electrolytes observed from FTO side at different gelation temperatures ((**a**,**h**) 30 °C, (**b**,**i**) 35 °C, (**c**,**j**) 40 °C, (**d**,**k**) 45 °C, (**e**,**l**) 50 °C, (**f**,**m**) 55 °C, and (**g**,**n**) 60 °C).

**Morphologies of cells and electrolytes.** Digital camera images of the fabricated cells and electrolytes taken from cells are shown in Figure 3a–g and Figure S1, which were gelled at different temperatures (30, 35, 40, 45, 50, 55, and 60 °C). It is worth noting that the color suddenly changes from yellow–green to orange at 50 °C. The reason is considered to be that the chemical bonding state around Cu atoms changed. The middle part of the electrolyte gelled at higher temperatures (50~60 °C) lacks integrity because the evaporated NMP diffuses from the middle to the surroundings during the gelation process. The evaporated NMP was adsorbed by the sealing material which was composed of Poly(alkylalkoxysiloxane), Poly(alkylsiloxane), silica, etc. Moreover, microphotographs of the electrolytes were observed from the FTO side, as shown in Figure 3h–n. The surface morphology of the electrolyte is changed from a smooth structure to a particle domain structure with increasing gelation temperature, suggesting that the gelled state of the electrolyte changes with temperature.

**Figure 4:**
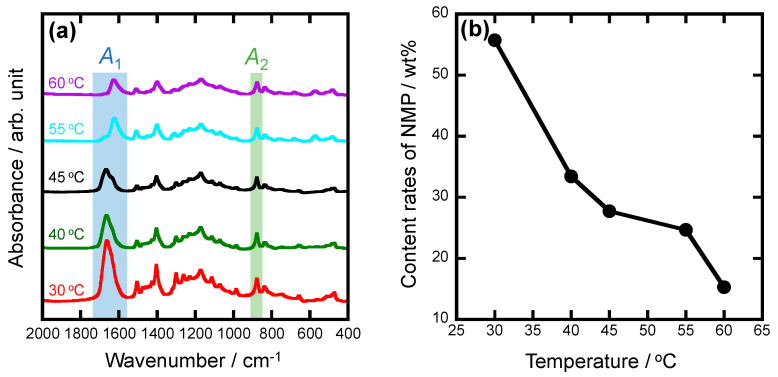
(**a**) FT-IR spectra and (**b**) content rates of NMP for the electrolytes at different gelation temperatures, where *A*_1_ is the absorption band due to C=O stretching vibration of NMP and *A*_2_ is the absorption band due to CF_2_ stretching vibration of PVDF-HFP.

**Chemical bonding states of electrolytes changing with gelation temperatures.** FT-IR spectra of the electrolytes at different gelation temperatures are shown in Figure 4a, in which *A*_1_ is the absorption band area (1740~1540 cm^−1^) due to C=O stretching vibration of NMP [36] and *A*_2_ is the absorption band area (900~860 cm^−1^) due to CF_2_ stretching vibration of PH [38,39]. The content rates of NMP for the electrolytes gelled at different temperatures were calculated by Equation (1). The content rates of NMP tend to decrease with increasing gelation temperature (Figure 4b), indicating that the NMP was gradually evaporated. Thus, the color of the electrolyte was changed with gelation temperature and 80 wt% NMP was evaporated at 60 °C. UV–Vis absorption spectra of the electrolytes gelled at 35 °C, 45 °C, and 55 °C are shown in Figure S2. Comparing the electrolytes gelled at 35 and 45 °C, there was a new absorption band caused by *d*-*d* transition due to Cu complex observed for the electrolytes gelled at 55 °C [40,41,42,43]. Thus, it indicated that the chemical bonding states of the electrolytes changed depending on gelation temperatures. The Cu complex was formed when the NMP was largely evaporated at the higher gelation temperature.

### 3.2. Electrochemical Performances of GPEs

**Figure 5:**
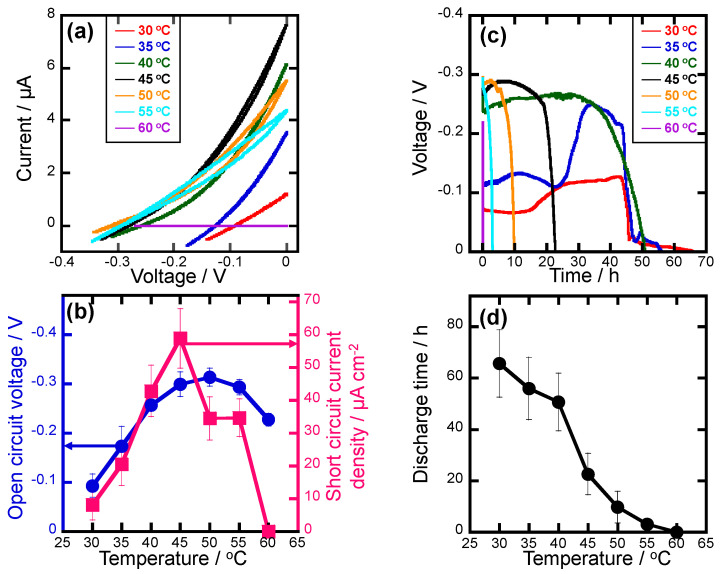
(**a**) Cyclic voltammetry and (**c**) chronoamperometry of the cells measured at different temperatures which are equal to the gelation temperatures. (**b**) Open−circuit voltages, short−circuit current densities, and (**d**) discharge times of the cells are obtained from CV and CP measurements.

**CV measurements.** CVs of the cells measured at different temperatures are shown in Figure 5a, where the measurement temperatures are equal to the gelation temperatures. The open-circuit voltages and short-circuit current densities were obtained from CV curves, which are shown in Figure 5b. The open-circuit voltages and short-circuit current densities show the similar tendency changing with the temperatures where they reached a maximum at around 45 °C. The open-circuit voltage of the STC system is the difference between the Fermi level of the semiconductor and the redox potential of the ions [8,44]. According to the Nernst equation, the redox potential increased with increasing temperature. Thus, the open-circuit voltages increased with the temperature changing from 30 °C to 45 °C. On the other hand, the concentration of Cu ions in the electrolyte increased with increasing temperature due to the gradual evaporation of NMP. As a result, the short-circuit current densities increased with the temperatures changing from 30 °C to 45 °C. The open-circuit voltages and the short-circuit current densities decreased with the temperatures changing from 45 °C to 60 °C because the internal resistances of cells increased with solidifying of electrolytes (Figure 3 and Figure S1). It was found that there was no faradaic current for the cell gelled and measured at 60 °C, indicating that the internal impedance of the electrolyte was very large. The temperature dependence of short-circuit current density (Arrhenius plot) in the range of 30~45 °C was plotted as shown in Figure S3. The activation energy was calculated to be 107 kJ mol^−1^ based on Equation (1), and that was smaller compared to the PEG600-based liquid electrolyte (150 kJ mol^−1^) in the STC system [7].

**CP measurements.** CP of the cells measured at different temperatures is shown in Figure 5c, where the measurement temperatures are equal to the gelation temperatures. As shown in Figure 5d, the discharge times of the cells tend to decrease with increasing temperature from 30 °C to 60 °C, which is consistent with the decreasing trend of NMP. On the other hand, it was found that when the diffusion and supply of Cu ions could not keep up with the charge transfer at the electrode/electrolyte interface, the discharge was terminated (the voltage reaches 0) for all cells. Thus, it indicates that the NMP plays an important role in the diffusion of Cu ions during the discharge process, NMP acts as a medium for Cu ion diffusion, so the decrease in NMP leads to a reduction in the discharge time. From the CP curves, there was a behavior of the temporary increase in voltage over time at 30 °C and 35 °C. The reason is considered to be that part of the free NMP molecules exist in the electrolytes gelled at 30 °C and 35 °C, and free NMP molecules were further evaporated during the discharge process causing the concentration of Cu ions in the electrolyte to increase, thereby temporarily increasing the voltage over time. According to the above results, the possible chemical bonding states for the electrolytes gelled at different gelation temperatures were considered and are shown in Figure S4. It is inferred that the Cu ions combined with PH and NMP to form the different chemical composite structures such as PH/NMP/Cu and NMP/Cu for the electrolyte gelled at lower temperatures (30~40 °C). Moreover, based on the shape of the CV, the charge transfer due to the chemical reaction was rate-limiting in the measurement range. As a result, slower charge transfer was caused at the electrode/electrolyte interface and the smaller short-circuit current densities were obtained for the electrolyte gelled at lower temperatures. Moreover, there was a large evaporation of NMP in the electrolyte gelled at higher temperatures (50~60 °C) that led to the formation of Cu complexes, which further caused larger internal resistance and lower short-circuit current densities and the color of electrolyte changes from yellow–green to orange at 50 °C. As for the electrolyte gelled at 45 °C, a well-balanced structure among the NMP molecules was formed to generate a high-efficiency conduction path of the Cu ions [29] and further cause the highest short-circuit current density.

**Electrochemical impedances and ion diffusion lengths.** To evaluate the electrochemical impedance and ion diffusion length, PEIS were also performed. The equivalent circuit was constructed using symmetric cells, in which the electrolyte was sandwiched between the FTO and FTO electrodes. The equivalent circuit was determined as shown in Figure S5a, where R1 is internal electrolyte resistance, R2 is charge transfer resistance at the FTO/electrolyte interface, CPE1 is electric double layer at the FTO/electrolyte interface, and Ws1 is Warburg element representative of ion diffusion. PEIS measurement of the cells at 35 °C, 40 °C, and 45 °C are shown in Figure S5b. Based on the equivalent circuit, the PEIS curves were fitted and the values of internal electrolyte resistance and Warburg element representative of ion diffusion (*W*) were obtained (Table 1). The ion conductivities (*σ*) of the cells were calculated through Equation (4) and are shown in Table 1, indicating that the σ values decreased with increasing temperatures from 35 °C to 45 °C because of the gradual decrease in the content rate of NMP.

**Diffusion coefficients.** The CV curves and oxidation and reduction peak currents of the cells changing with scan rate are shown in Figure S6. The CV curves changing with different scan rates were measured to calculate the diffusion coefficients (*D*) through Equation (3). The measured *D* values were close to the previously reported diffusion coefficient of PVDF-Type GPE [45]. The *D* value of Cu ions in the electrolyte gelled at 45 °C showed a higher value than that at 35 and 40 °C (Table 1), indicating that a well-balanced structure among the NMP molecules was formed to generate a high-efficiency diffusion path of the Cu ions in the electrolyte gelled at 45 °C.

**Ion diffusion lengths.** Based on the values of *D* and *W* obtained from PEIS measurement, the ion diffusion lengths (*l*) were calculated through Equation (5) and are shown in Table 1, indicating that the *l* values decreased with increasing temperatures from 35 °C to 45 °C. This difference in *l* might be caused by the content rates of NMP in the gelled electrolytes; NMP as a medium plays a very important role in the diffusion of Cu ions. Thus, the higher content rate of NMP in the electrolyte gelled at 35 °C results in the larger *l* value of 0.37 µm. In contrast, the *l* value of Cu ions in the PEG600-based liquid electrolyte was 70 µm at 80 °C [46].

**Recovery behaviors.** We found that the once-discharged cells could not discharge again, even leaving it in an open-circuit (recovery) state for 5 h (Figure S7) or more than two days. The reason was considered to be that the *l* values (0.14~0.37 μm) were too small, and the distance between the electrodes (85 μm) is greater than two times the *l* value for each cell. Thus, the diffusion layers approximately equal to *l* values existing on the surface of both electrodes were not overlapped, so the ion diffusion between the two electrodes was hindered. Furthermore, in order to evaluate the other reasons, the FT-IR and UV–Vis absorption spectra of the cells before and after discharge at 45 °C are shown in Figure S8a,b. As for the results of FT-IR spectra, compared to the electrolyte before discharge, the C=O stretching vibration of NMP shifts to the lower wavenumber side for the discharged electrolyte. This indicated that the chemical bonding between Cu ions and C=O (NMP) became stronger in the discharged electrolyte. Moreover, the new absorption band caused by d-d transition due to the Cu complex was also observed in the discharged electrolyte according to the UV–Vis spectra [40,41,42,43]. The Cu complex was formed in the electrolyte of the discharged cell so that the diffusion of the Cu ions is inhibited. Therefore, these are the possible reasons why we observed that the once-discharged cells could not discharge again, even leaving it in an open-circuit state for a long time. In the future, we will continue to improve the electrochemical properties of this GPE so that it can successfully recover power generation ability.

## 4. Conclusions

The PH-based GPEs were synthesized under different gelation temperatures. It was found that the PH-based GPE gelled at 45 °C showed higher open-circuit voltage (−0.3 V), short-circuit current density (59 μA cm^−2^), and diffusion coefficient (7.82 × 10^−12^ m^2^ s^−1^), indicating that a well-balanced structure among the NMP molecules was formed to generate a high-efficiency conduction path of the Cu ions. The PH-based GPE gelled more than 45 °C had poor electrochemical performance and thermal stability. The reason was considered to be that there was a large evaporation of NMP in the electrolyte gelled at higher temperatures to form the Cu complex which caused larger internal resistance and lower short-circuit current density and open-circuit voltage. Since the evaporation of NMP at higher temperatures is inevitable, determining how to improve the high-temperature stability of the PH-based GPE is a future research topic. On the other hand, Cu ion concentration as another important factor needs to be considered. In the future, we will discuss the Cu ion concentration as a variable in detail and plan to report it as a separate paper.

## Figures and Tables

**Table 1:** Ionic conductivity (*σ*), diffusion coefficient (*D*), Warburg element (*W*), and ion diffusion length (*l*) of the cell at 35 °C, 40 °C, and 45 °C.

Gelation Temperature (°C)	*σ* (×10^−2^, cm^−1^ Ω^−1^)	*D* (×10^−12^, m^2^ s^−1^)	*W* (×10^−2^, s)	*l* (μm)
35	1.73	7.61	1.80	0.37
40	1.72	5.31	0.49	0.16
45	1.68	7.82	0.24	0.14

## Data Availability

Data are contained within the article and Supplementary Materials.

## Supplementary Materials

The following supporting information can be downloaded at: https://www.mdpi.com/article/10.3390/polym16121732/s1, Figure S1: Digital camera images of the electrolytes; Figure S2: UV–Vis absorption spectra of the electrolytes; Figure S3: Temperature dependence of short-circuit current density (Arrhenius plot) to calculate activation energy; Figure S4: Graphic images of the possible states for the electrolytes gelled at different gelation temperatures; Figure S5: Equivalent circuit using symmetric FTO/FTO cells and PEIS measurement; Figure S6: Cyclic voltammetry, oxidation and reduction peak current of the cell changing with scan rates; Figure S7: Discharge (chronoamperometry) and recovery of the cells; Figure S8: FT-IR and UV–Vis absorption spectra of the cells before and after discharge.
