# Supplementary material for: Preparation and Evaluation of PVDF-HFP-Based Gel Electrolyte for Ge-Sensitized Thermal Cell
Source: Polymers (Basel). 2024 Jun 18;16(12):1732. doi: 10.3390/polym16121732 (PMC11207463; doi:10.3390/polym16121732)
Supplement: Supplementary file 1 [file polymers-16-01732-s001.zip › polymers-3025659-supplementary.pdf]

*Electronic Supplementary Materials*

# Preparation and Evaluation of PVDF-HFP Based Gel Electrolyte for Ge-sensitized Thermal Cell


Yadong Chai [1], Sachiko Matsushita [1,2,*]

[1] Department of Materials Science and Engineering, Tokyo Institute of Technology, 4259 Nagatsuta-cho, Midori-ku, Yokohama, Kanagawa 226-8501 Japan

[2] elleThermo, Ltd., Tokyo Institute of Technology, Yokohama 226-8503, Japan

*** Author to whom correspondence should be addressed:**

Tel: +81-45-924-5163, E-mail: matsushita.s.ab@m.titech.ac.jp

## *Experimental procedure S1*

The content rate of NMP in the prepared electrolyte was calculated through **Equation (S1)**, where the added weight of NMP, PVDF-HFP, CuCl and $CuCl_2$ were 4, 1, 0.16 and 0.21 g respectively.

$$\frac{4}{4+1+0.16+0.21} \times 100\% = 74.5 \text{ wt\%} \tag{S1}$$

## *Figure S1*

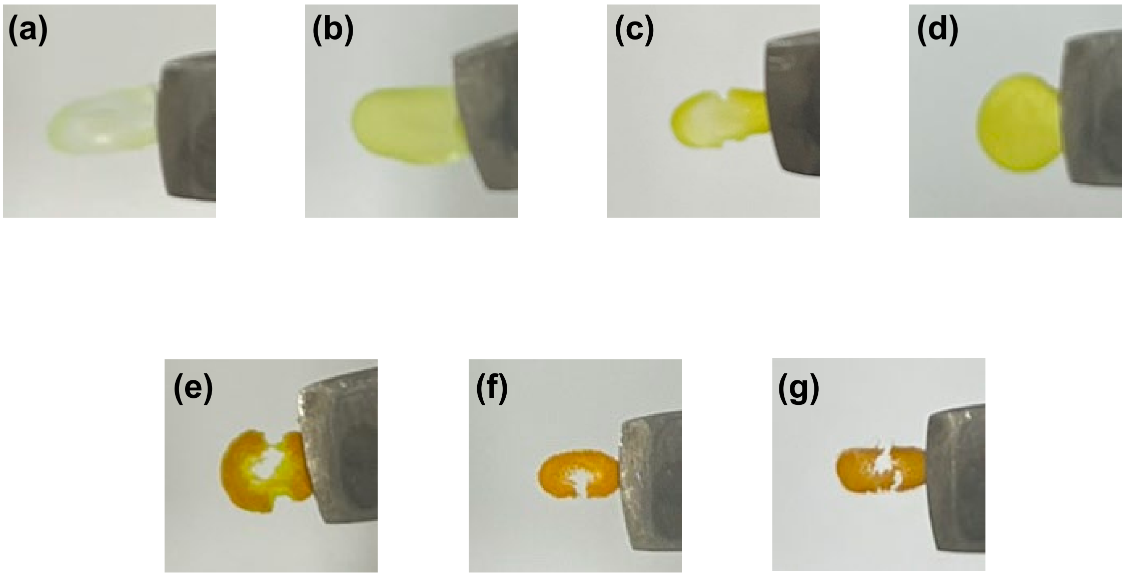


**Figure S1.** Digital camera images of the electrolytes at different gelation temperatures ((a) 30$^{o}$C, (b) 35$^{o}$C, (c) 40$^{o}$C, (d) 45$^{o}$C, (e) 50$^{o}$C, (f) 55$^{o}$C and (g) 60$^{o}$C ).

*Figure S2*

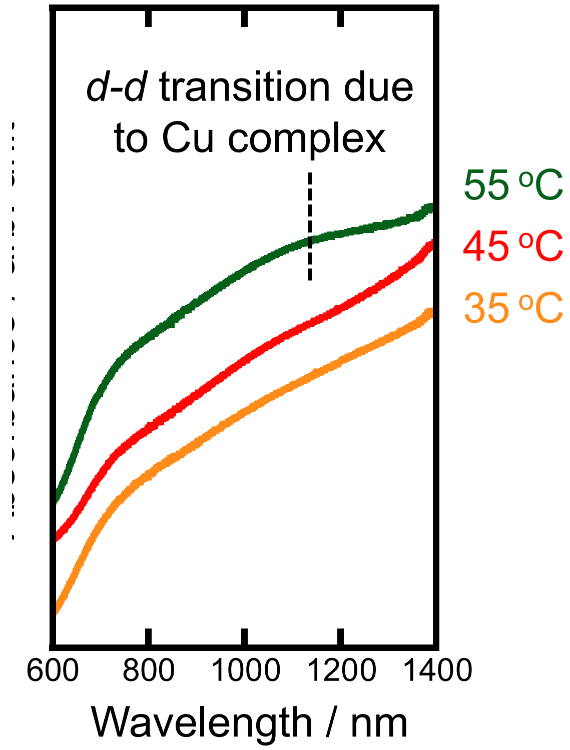


**Figure S2**. UV-vis absorption spectra of the electrolytes measured at 35 °C (orange line), 45 °C (red line) and 55 °C (green line).

## *Figure S3*

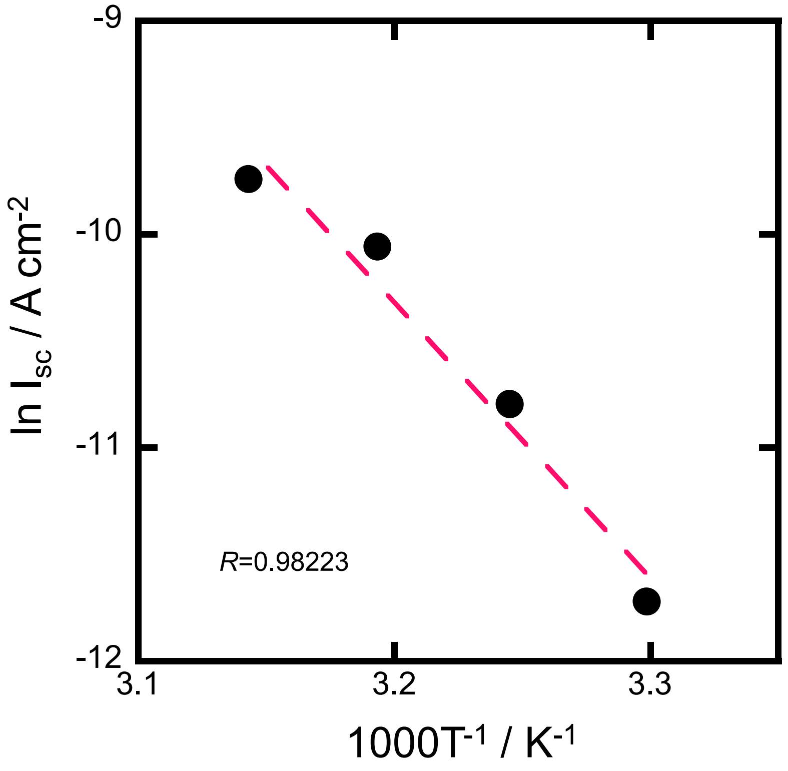


**Figure S3**. Temperature dependence of short circuit current density (Arrhenius plot) to calculate activation energy which was 107 kJ $mol^{-1}$.

## *Figure S4*

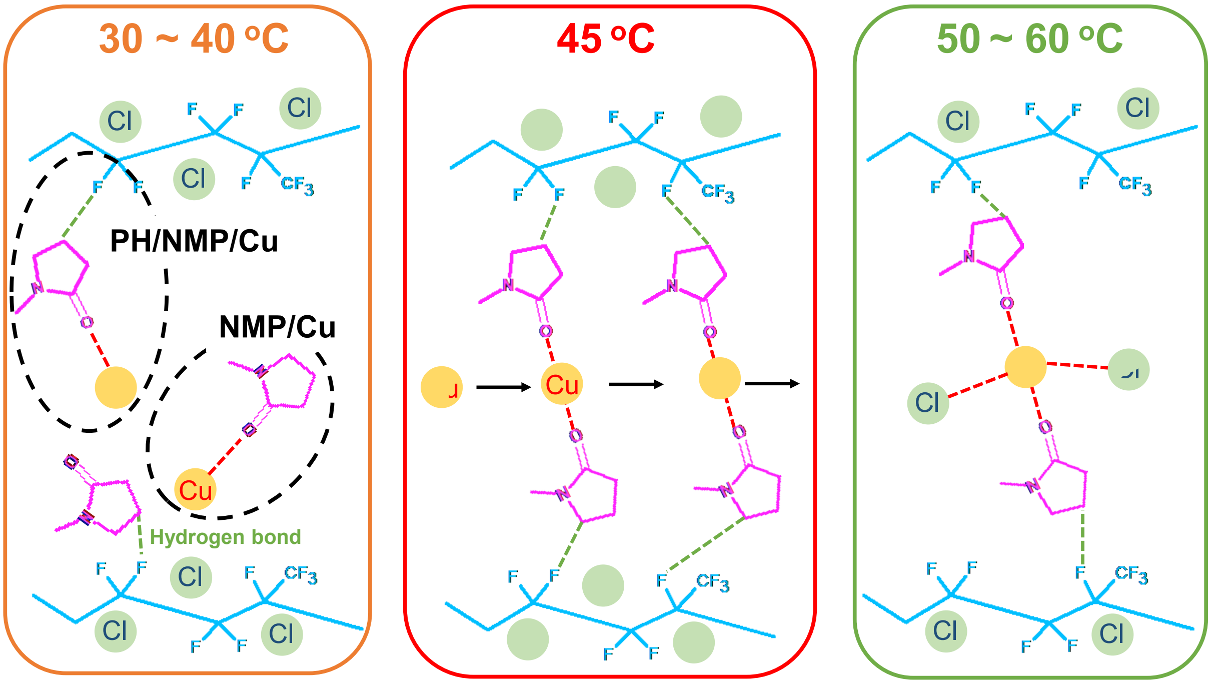


**Figure S4**. Graphic images of the possible states for the electrolytes gelled at different gelation temperatures.

## *Figure S5*

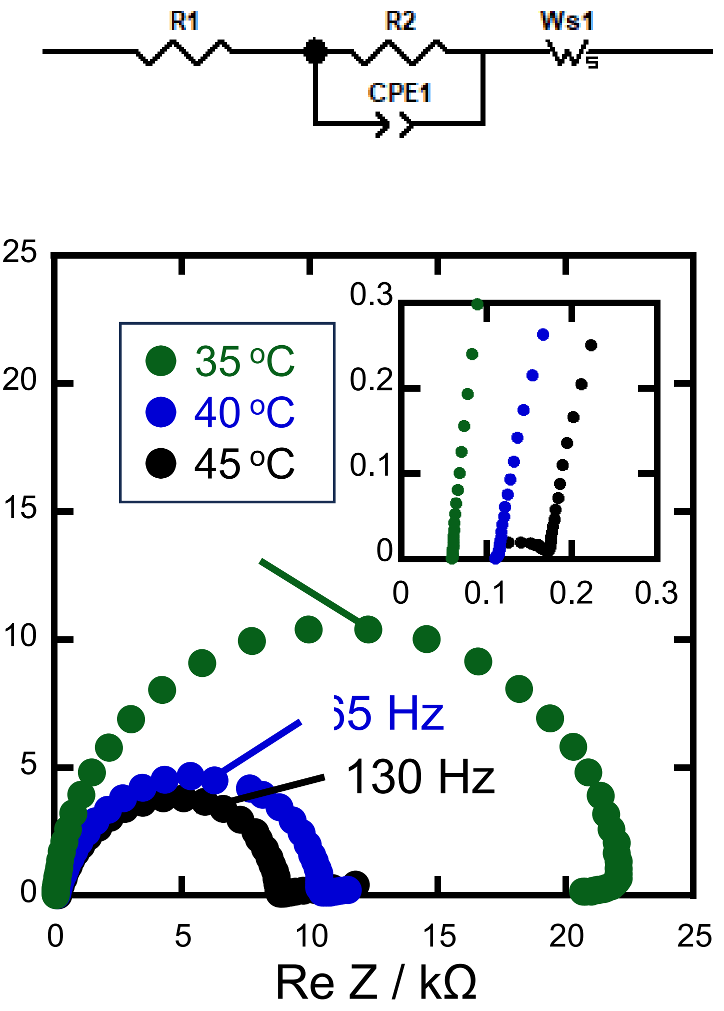


**Figure S5**. (a) Equivalent circuit using symmetric FTO/FTO cells. R1: internal electrolyte resistance; R2: charge transfer resistance at the FTO/electrolyte interface; CPE1: electric double layer at the FTO/electrolyte interface and Ws1: Warburg element representative of ion diffusion, and (b) PEIS measurement of the cells at 35 °C, 40 °C and 45 °C.

## *Figure S6*

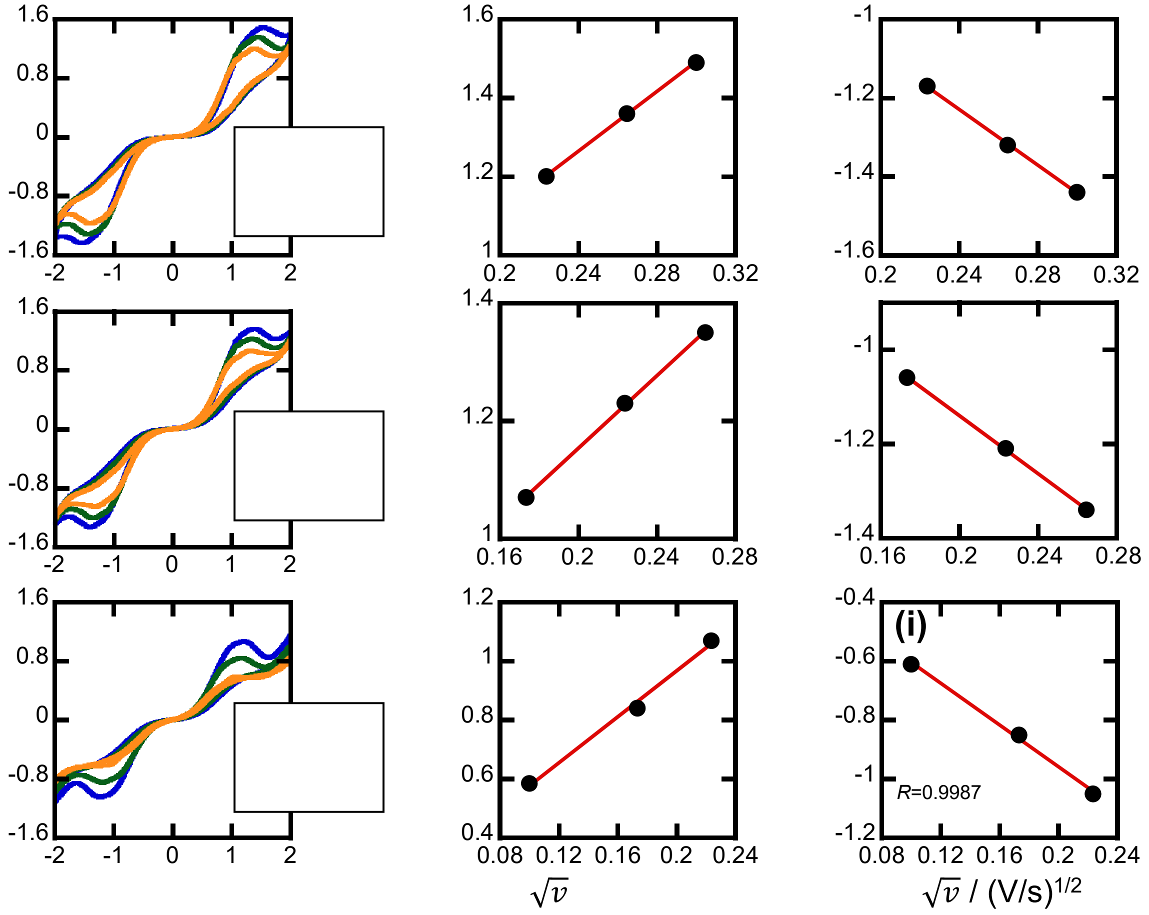


**Figure S6**. (a, d, g) Cyclic voltammetry, (b, e, h) oxidation and (c, f, i) reduction peak current of the cell at (a~b) 35 °C, (d~f) 40 °C and (g~i) 45 °C changing with scan rate.

## *Figure S7*

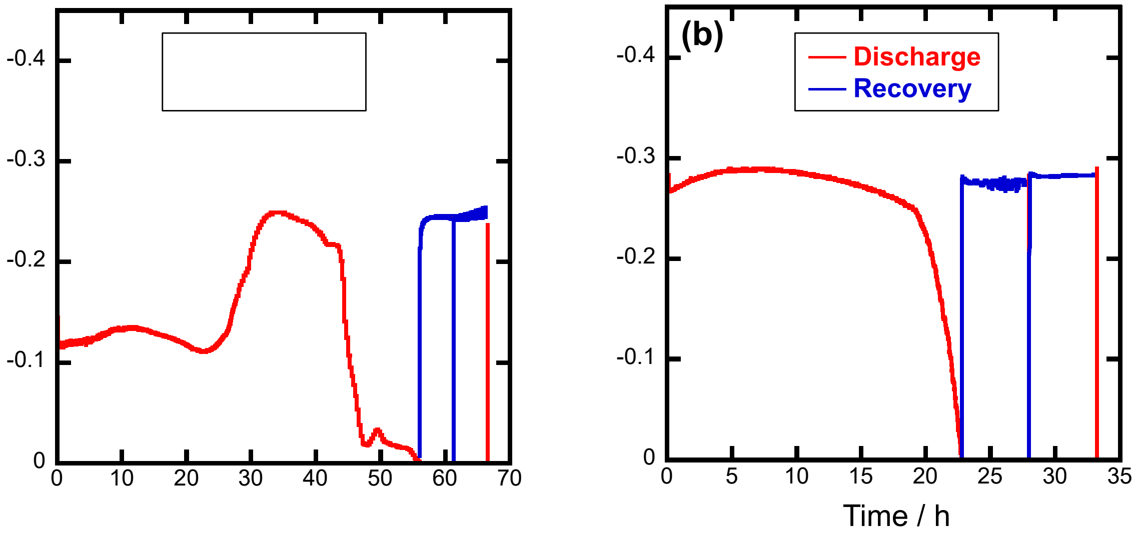


**Figure S7**. Discharge (chronoamperometry) and recovery of the cells were measured at (a) 35 °C and (b) 45 °C which are equal to the gelation temperatures, where the discharge current is 200 nA, the recovery time was 5 h and the trial number of discharges was 3.

## *Figure S8*

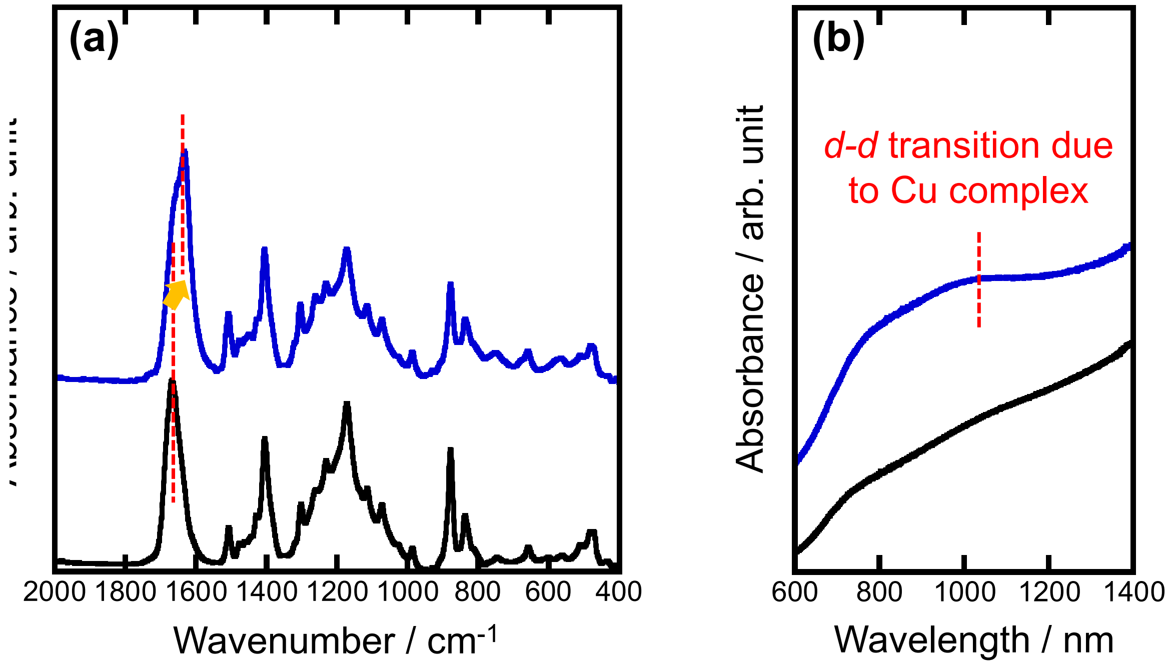


**Figure S8**. (a) FT-IR and (b) UV-vis absorption spectra of the cells before (indicated by black line) and after discharge (indicated by blue line) at 45 °C.
